# The add-on effects of Danhong injection among patients with ischemic stroke receiving Western medicines: A systematic review and meta-analysis

**DOI:** 10.3389/fphar.2022.937369

**Published:** 2022-08-23

**Authors:** Yu Ma, Ke Deng, Jiali Liu, Bin Ma, Fan Mei, Wen Hui, Xiaochao Luo, Minghong Yao, Yanmei Liu, Xuan Qin, Xu Zhou, Kang Zou, Ling Li, Xin Sun

**Affiliations:** ^1^ Chinese Evidence-Based Medicine Center, West China Hospital, Sichuan University, Chengdu, Sichuan, China; ^2^ NMPA Key Laboratory for Real World Data Research and Evaluation in Hainan, Chengdu, Sichuan, China; ^3^ Sichuan Center of Technology Innovation for Real World Data, Chengdu, Sichuan, China; ^4^ Evidence-Based Medicine Center, School of Basic Medical Sciences, Lanzhou University, Lanzhou, Gansu, China; ^5^ Department of Science and Technology, West China Hospital, Sichuan University, Chengdu, Sichuan, China; ^6^ Evidence-Based Medicine Research Center, School of Basic Science, Jiangxi University of Traditional Chinese Medicine, Nanchang, Jiangxi, China

**Keywords:** Danhong injection, ischemic stroke, meta-analysis, systematic review, randomized controlled trial

## Abstract

**Background:** Danhong injection is widely used for treating ischemic stroke in China. However, its effects on ischemic stroke patients when given along with Western medicines (i.e., the add-on effect) were not well-established.

**Methods:** We searched PubMed, Embase, Cochrane Central Register of Controlled Trials (CENTRAL), and three Chinese databases from inception to 20 July 2020 to identify randomized controlled trials (RCTs) that assessed the effects of Danhong injection as add-on therapy in patients with ischemic stroke. Pairs of trained reviewers independently screened for eligible studies, assessed risk of bias, and extracted the data. The outcomes were the National Institutes of Health Stroke Scale Score (NIHSS), Barthel index, activities of daily living (ADL), total cholesterol, and homocysteine (Hcy).

**Results:** Sixty-seven RCTs of 6594 patients with varying risk of bias were included. Compared with Western medicine alone, the addition of Danhong injection to Western medicine significantly lowered the NIHSS score (45 RCTs with 4565 patients; MD −4.21, 95% CI −4.96 to −3.46), total cholesterol (10 trials with 1019 patients; MD −1.14 mmol/L, 95% CI −1.57 to −0.72), and Hcy (four trials with 392 patients; MD −3.54 μmol/L, 95% CI −4.38 to −2.07). The addition of Danhong also increased the Barthel index (14 trials with 1270 patients; MD 8.71, 95% CI 3.68–13.74) and ADL (12 trials with 1114 patients; MD 14.48, 95% CI 9.04–19.92) scores. Subgroup analyses showed differential effects in the average cerebral blood flow rate by mean age of patients (<60 years: MD 0.74 cm/s, 95% CI 0.29–1.19; ≥60 years: MD 4.09 cm/s, 95% CI 2.02–6.16; interaction *p* = 0.002) and the NIHSS score by type of baseline Western medicines (interaction *p* < 0.00001).

**Conclusion:** The addition of Danhong injection to Western medicine may improve neurological function, self-care ability, and blood lipid level of ischemic stroke patients. However, given most included trials with unclear risk of bias, current evidence is not definitive, and more carefully designed and conducted trials are warranted to confirm our findings.

**Systematic Review Registration**: [https://www.crd.york.ac.uk/PROSPERO/], identifier [CRD42022298628].

## Introduction

Ischemic stroke, defined as all thromboembolic and atherosclerotic events resulting in compromised blood flow to cerebral tissue and subsequent infarction (Collaborators, 2019), is a leading cause of death and long-term disability worldwide. Current recommendations for patients with ischemic stroke include antiplatelet, anticoagulants, volume expansion, vasodilators, neuroprotective agents, intravenous thrombolysis, and mechanical thrombectomy (Powers et al., 2019). In spite of these, patients with ischemic stroke continue to suffer from a high risk of disability and recurrence, and add-on treatments are often necessary (van der Meij and Wermer, 2021).

Danhong injection is a Chinese patent medicine extracted from *Salvia miltiorrhiza* Bunge (Lamiaceae; *Salviae miltiorrhizae* radix et rhizoma) (Danshen in Chinese) and *Carthamus tinctorius* L (Compositae; carthami oleum raffinatum) (Honghua in Chinese), which improve microcirculation, prevent platelet aggregation, decrease plasma viscosity, and boost the activity of fibrinogen dissolved. Danhong injection has been widely used for cardiovascular and cerebrovascular diseases (Xu et al., 2018; Feng et al., 2019). Evidence from randomized controlled trials (RCTs) with small sample sizes showed that the combination of Danhong injection and Western medicine was effective in improving total efficiency and hemodynamic outcomes among ischemic stroke patients (Liu et al., 2019; Li et al., 2020; Liu and Jin, 2020). Three systematic reviews also found that, compared with other Chinese botanical drug injections (e.g., Shuxuening injection), Danhong injection appeared to be beneficial in total clinical effectiveness rate and neurologic impairment (Wang et al., 2017; Liu et al., 2018; Liu et al., 2019b). However, these reviews included a relatively small number of RCTs and were limited with improper comparisons, inappropriate outcome selection, and analysis, as a result of which the resulting findings and recommendations were not definitive.

Most importantly, Danhong injection is routinely used as an add-on therapy for patients with ischemic stroke receiving Western medicine; its add-on effects on ischemic stroke patients still need further systematic assessment to inform current practice. Therefore, we conducted this systematic review and meta-analyses, which included a significantly larger number of newly published RCTs.

## Methods

We followed the preferred reporting items for systematic reviews and meta-analyses (PRISMA) statement (Page et al., 2021) to conduct and report this systematic review and meta-analysis. This protocol was registered with the International Prospective Register of Systematic Reviews (CRD42022298628).

### Eligibility criteria

We included randomized controlled trials (RCTs) that compared the efficacy and safety of Danhong injection combined with Western medicine therapy versus Western medicine alone in patients with ischemic stroke. Western medicine therapy in our study refers to the western standard of care, which contained a range of Western drugs, interventional therapy, and surgical treatment. There was no limitation on the dosages and courses of treatment. Eligible studies explicitly reported outcome data on the National Institute of Health Stroke Scale (NIHSS), the Fugl–Meyer assessment, the Barthel index, activities of daily living (ADL), intima-media thickness (IMT), cerebral blood flow, average cerebral blood flow rate, total cholesterol, triglycerides, high-density lipoprotein (HDL), low-density lipoprotein (LDL), homocysteine (Hcy), D-dimer, and adverse drug reactions (ADRs)/adverse drug events (ADEs). We excluded RCTs that involved other Chinese botanical drugs.

### Literature search

We searched PubMed, Embase, Cochrane Central Register of Controlled Trials (CENTRAL), the China National Knowledge Infrastructure (CNKI) database, the Wanfang database, and the Chinese Scientific Journal Database to identify relevant studies from inception to 20 July 2020. The subject terms (e.g., MeSH terms) and free-text words were used to search for potentially eligible studies, and language restriction was imposed in English and Chinese. The specific retrieval strategy was provided in Supplementary Appendix S1.

### Study process

Two reviewers (YM and KD) independently screened titles/abstracts and full texts for eligibility, assessed risk of bias, and collected data from each eligible study using a prespecified form. Reviewers dealt with discrepancies through discussion or, if required, adjudication by a third researcher (LL).

### Risk of bias assessment

We used the modified Cochrane Risk of Bias Assessment Tool to assess the risk of bias of included RCTs (Higgins et al., 2011). The items contained random sequence generation (selection bias), allocation concealment (selection bias), blinding of patients and personnel (performance bias), blinding of outcome assessment (detection bias), incomplete outcome data (attrition bias), selective outcome reporting (reporting bias), and other sources of bias. The judgment for each item included a low/high/unclear risk of bias.

### Data extraction

We collected the following information from included RCTs: general study characteristics (first author, year of publication, and total number of patients randomized); patient characteristics (age and sex); intervention and control characteristics [medications used across groups (baseline treatment), details of Danhong injection treatment and the control group (dose and type of Western medicines used), and course of treatment]; outcome data (mean and standard deviation of continuous variables, and number of patients with adverse drug reactions/adverse drug events in each group).

### Statistical analysis

Reviewer Manager 5.3 (Cochrane Collaboration, Oxford, United Kingdom) was used to analyze and compare the efficacy outcomes of the Danhong injection groups versus the control groups. The mean difference (MD) was calculated for continuous variables; the effects of each outcome were estimated with 95% confidence intervals (95% CIs). We used Cochran’s chi-squared test and the *I* ^*2*^ statistic to examine statistical heterogeneity among RCTs. Meta-analysis was calculated by a random-effects model.

We explored sources of heterogeneity with three prespecified subgroup hypotheses: length of treatment (≤2 vs. > 2 weeks; larger effect in trials with longer treatment), mean age of patients (<60 vs. ≥ 60 years old; larger effect in trials with younger patients), and types of baseline Western medicine treatment (conventional therapy vs. intravenous thrombolysis vs. anti-platelet aggregation vs. dilation of blood vessels vs. statins vs. neuroprotective agents vs. other therapies).

In addition, we used the funnel plot and Egger’s test for examining publication bias (Egger et al., 1997). We also used the Duval and Tweedie trim-and-fill method for exploring the influence of a single trial on publication bias (Duval and Tweedie, 2000).

## Results

### Search results

**FIGURE 1:**
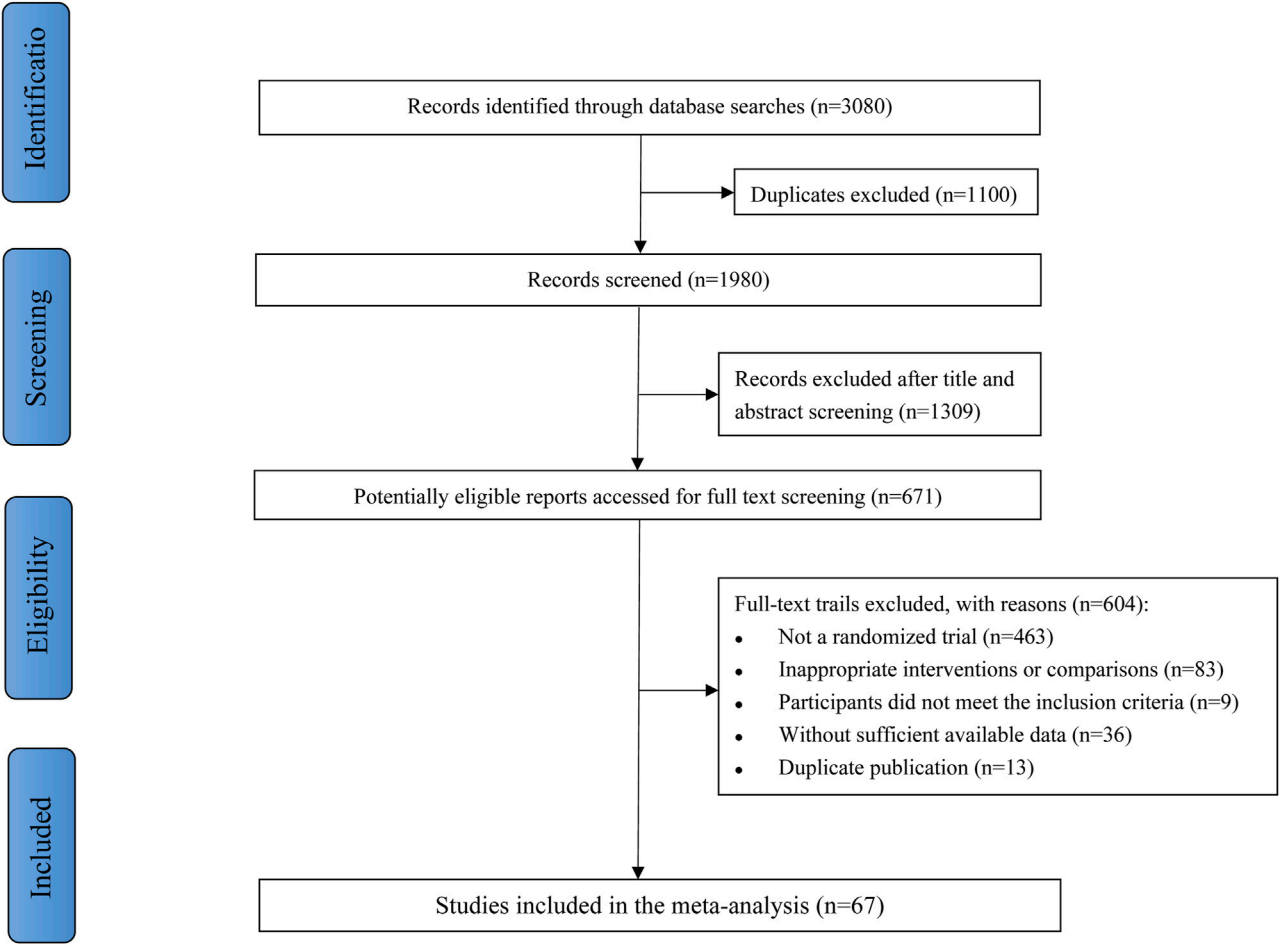
Flow chart of study selection.

Figure 1 described the study selection process. Our search retrieved 3,080 citations during the initial detection by the search strategy. First, 1,100 duplicates were removed, and 1309 studies were excluded after screening titles and abstracts. Of 671 studies included for full further text screening, 604 studies were excluded for the following reasons: not a randomized trial (*n* = 463), inappropriate interventions or comparisons (*n* = 83), participants did not meet the inclusion criteria (*n* = 9), without sufficient available data (*n* = 36), or duplicate publication (*n* = 13). Finally, 67 RCTs were eligible for meta-analysis.

### Patient characteristics

These 67 RCTs (Liu and Huang 2010; Xue eand Li, 2010; Ren et al., 2011; Luo et al., 2012; Shen, 2012; Su et al., 2012; Zou et al., 2013; Liang et al., 2014; Song and Yang 2014; Yin, 2014; Fan et al., 2015; Li, 2015; Ma et al., 2015; Zhang, 2015; Cao and Mei 2016; Feng et al., 2016; Wang, 2016; Wang et al., 2016; Wu and Bao 2016; Zeng et al., 2016; Li Q. et al., 2017; Liu F. et al., 2017; Li Y. et al., 2017; Liu Z. et al., 2017; Chen, 2017; Chen and Shi., 2017; Li, 2017; Liu, 2017; Ma, 2017; Ou and Liu 2017; Pen, 2017; Qiu et al., 2017; Wei et al., 2017; Yun et al., 2017; Zhang, 2017; Yang and Liu 2018a; Yang et al., 2018b; Dai, 2018; Fan et al., 2018; Ge, 2018; Li et al., 2018; Liu, 2018; Luo, 2018; Lv et al., 2018; Shi, 2018; Yang, 2018; Zhang et al., 2018; Liu Q. et al., 2019; Cao et al., 2019; Chai et al., 2019; Chen et al., 2019; Cheng, 2019; Liu Y. et al., 2019; Deng, 2019; Jin et al., 2019; Li and Tian 2019; Yuan, 2019; Zhang, 2019; Cao, 2020; Huo et al., 2020; Jiang et al., 2020; Jing and Yu, 2020; Kang et al., 2020; Li et al., 2020; Liu and Jin, 2020; Zhu and Qi 2020; Zhuang and Zhao., 2020) comprised 3293 patients in the combination treatment group and 3301 patients in the control group. The mean age of included patients ranged from 42.2 to 71.5 years, and the sample size ranged from 40 to 300 patients. All RCTs were conducted among Chinese populations in China. Patients in the combination treatment group received Danhong injection of 10–40 ml once or twice daily. There was no significant difference between the experiment group and the control group in baseline characteristics (Table 1).

**TABLE 1:** Baseline characteristics of included randomized controlled trials.

Author (year)	Phase of disease	Sample size EG/CG	Mean age (year)		Intervention		Dosage of danhong injection	Course	Outcome
						EG				CG	EG	CG
Cao (2020)	Acute phase	49/49	63.11	62.47	rt-PA, DHI	rt-PA	Once daily, 20 ml	14d	(1) (12) (14)
Cheng (2019)	NR	20/20	58.40	56.10	Edaravone, DHI	Edaravone	Once daily, 30 ml	14d	(14)
Zhang (2019)	NR	55/55	53.80	53.20	Clopidogrel, DHI	Clopidogrel	Once daily, 30 mg	28d	(14)
Deng (2019)	NR	50/50	63.77	63.28	Edaravone, DHI	Edaravone	Once or twice daily, 20–40 ml	14d	(1)
Li et al. (2017a)	Acute phase	40/40	66.50	66.50	Edaravone, DHI	Edaravone	Once daily, 30 ml	14d	(1) (4) (14)
Li (2017)	Acute phase	30/30	61.48	62.03	Ozagrel Sodium, DHI	Ozagrel Sodium	Once daily, 40 ml	14d	(1) (8) (9) (10) (11)
Wang (2016)	Acute phase	35/37	NR	NR	Aspirin, DHI	Aspirin	Once daily, 40 ml	14d	(3) (14)
Zhang (2015)	Acute phase	50/50	58.60	60.30	Vinpocetine, DHI	Vinpocetine	Once daily, 30 ml	14d	(1)
Yin (2014)	Acute phase	60/60	52.80	53.10	Hyperbaric oxygen, DHI	Hyperbaric Oxygen	Twice daily, 20 ml	56d	(1)
Shen (2012)	Acute phase	36/36	NR	NR	Edaravone, DHI	Edaravone	Once daily, 30 ml	14d	(1) (4) (14)
Zhuang and Zhao, (2020)	NR	56/56	61.11	61.15	CT, DHI	CT	Once daily, 30 ml	14d	(1) (14)
Liu (2018)	NR	83/83	NR	NR	Edaravone, DHI	Edaravone	Once daily, 20–40 ml	14d	(1) (14)
Shi (2018)	Acute phase	40/40	64.00	65.00	Alprostadil, DHI	Alprostadil	Once daily, 40 ml	14d	(1) (4)
Yang (2018)	Acute phase	47/47	NR	NR	Edaravone, DHI	Edaravone	Once daily, 40 ml	14d	(1) (3) (8) (9)
Ma (2017)	NR	40/42	60.01	65.26	CT, DHI	CT	Once daily, 20 ml	14d	(1) (2) (13)
Liu and Huang, (2010)	Acute phase	44/43	63.70	64.40	Fasudil hydrochloride, DHI	Fasudil hydrochloride	Once daily, 30 ml	14d	(4) (14)
Chen (2017)	NR	32/32	53.12	52.93	CT, DHI	CT	Once daily, 30 ml	7d	(8)
Zhang et al. (2018)	Acute phase	54/54	61.80	60.20	Alprostadil, DHI	Alprostadil	Once daily, 30 ml	14d	(1) (14)
Zhang (2017)	Acute phase	45/45	68.90	69.30	Edaravone, DHI	Edaravone	Twice daily, 20 ml	14d	(14)
Pen (2017)	NR	39/39	NR	NR	Atorvastatin calcium, DHI	Atorvastatin calcium	Once daily, 10 ml	14d	(5)
Fan et al. (2018)	Acute phase	35/35	49.32	50.45	CT, DHI	CT	Once daily, 40 ml	14d	(1) (4)
Li et al. (2017b)	Acute phase	48/48	55.76	56.37	Clopidogrel, DHI	Clopidogrel	Once daily, 30 ml	14d	(1) (4) (14)
Jiang et al. (2020)	Acute phase	60/60	60.47	59.82	rt-PA, Sodium heparin, DHI	rt-PA, Sodium heparin	Once daily, 20 ml	14d	(1) (5) (14)
Liu et al. (2019a)	Acute phase	56/56	56.91	55.73	Edaravone, DHI	Edaravone	Once daily, 20 mg	14d	(2) (3) (6) (7)
Liu (2017)	NR	30/30	60.24	58.49	CT, DHI	CT	Once daily, 30 ml	14d	(13)
Ge (2018)	NR	37/37	62.13	61.52	Atorvastatin calcium, DHI	Atorvastatin calcium	Once daily, 20 ml	14d	(1) (14)
Yun et al. (2017)	Acute phase	31/31	69.50	68.20	Aspirin, DHI	Aspirin	Once daily, 30 ml	14d	(7)
Liu et al. (2019b)	Acute phase	33/32	41.84	42.53	Butylphthalide soft capsules, DHI	Butylphthalide soft capsules	Once daily, 40 ml	14d	(1)
Dai (2018)	Acute phase	47/47	56.42	56.43	Aspirin, nimodipine, DHI	Aspirin, nimodipine	Twice daily, 40 ml	15d	(1) (4) (8) (9)
Yang (2018)	Acute phase	39/39	64.08	63.22	Atorvastatin calcium, DHI	Atorvastatin calcium	Once daily, 20 ml	14d	(1) (3)
Li (2015)	Acute phase	105/105	71.30	71.60	Edaravone, DHI	Edaravone	Once daily, 30 ml	14d	(1) (8) (9)
Li et al. (2020)	Acute phase	52/52	68.34	68.58	3-n-butylphthalide, DHI	3-n-butylphthalide	Once daily, 30 ml	14d	(1) (7)
Liu (2017)	Acute phase	54/55	56.83	56.94	CT, DHI	CT	Once daily, 20 ml	14d	(1)
Luo (2018)	Acute phase	56/56	65.40	65.10	CT, DHI	CT	Twice daily, 30 ml	28d	(2) (14)
Kang et al. (2020)	Acute phase	65/60	55.42	54.95	Tirofiban hydrochloride, clopidogrel, aspirin, DHI	Tirofiban hydrochloride, clopidogrel, aspirin	Once daily, 20 ml	14d	(1) (4) (14)
Yang (2018)	Acute phase	58/58	57.86	57.09	Clopidogrel, DHI	Clopidogrel	Once daily, 30 ml	14d	(1) (14)
Liu and Jin (2020)	Acute phase	71/71	63.24	61.97	Edaravone, DHI	Edaravone	Once daily, 20 ml	14d	(1) (3) (7)
Wang (2016)	Acute phase	56/56	NR	NR	CT, DHI	CT	Once daily, 40 mg	168d	(1)
Yuan (2019)	Acute phase	38/38	65.50	65.39	Edaravone, DHI	Edaravone	Once daily, 40 ml	14d	(1) (2) (3) (12)
Su et al. (2012)	Acute phase	38/37	61.58	63.01	CT, DHI	CT	Once daily, 40 ml	14d	(8) (9) (11) (14)
Luo et al. (2012)	Acute phase	90/90	NR	NR	Ozagrel Sodium, DHI	Ozagrel Sodium	Once daily, 40 ml	14d	(1)
Zou et al. (2013)	Acute phase	40/40	58.70	57.90	Vinpocetine, DHI	Vinpocetine	Once daily, 30 ml	14d	(1)
Liang et al. (2014)	Acute phase	45/45	53.50	52.60	CT, DHI	CT	Once daily, 30 ml	14d	(4)
Ma et al. (2015)	Acute phase	45/45	65.37	65.64	CT, DHI	CT	Once daily, 20 ml	28d	(2) (3)
Fan et al. (2015)	NR	45/45	62.30	61.50	CT, DHI	CT	Once daily, 20 ml	28d	(8) (9) (10) (11)
Zeng et al. (2016)	Acute phase	55/55	63.00	63.30	Edaravone, DHI	Edaravone	Once daily, 20 ml	15d	(3)
Feng et al. (2016)	Acute phase	20/20	59.82	60.50	rt-PA, DHI	rt-PA	Once daily, 20 ml	14d	(3) (13)
Ou and Liu, (2017)	Acute phase	60/60	58.10	57.20	CT, DHI	CT	Once daily, 20 ml	14d	(1) (4) (12)
Wu and Bao. (2016)	Acute phase	49/49	67.40	68.10	Alprostadil, edaravone, aspirin, DHI	Alprostadil, edaravone, aspirin	Once daily, 40 ml	14d	(1) (3) (12) (14)
Chen (2017)	Acute phase	40/40	67.34	66.46	CT, DHI	CT	Once daily, 20 ml	14d	(2) (14)
Qiu et al. (2017)	Acute phase	58/58	70.69	69.88	CT, DHI	CT	Once daily, 30 ml	14d	(1) (4) (8) (9)
Liu (2017)	Acute phase	48/48	52.33	52.05	CT, DHI	CT	Once daily, 20 ml	14d	(1)
Wei et al. (2017)	Acute phase	47/47	62.60	60.73	CT, DHI	CT	Once daily, 20 ml	14d	(1) (14)
Jing and Yu., (2020)	Acute phase	44/44	62.70	63.20	CT, DHI	CT	Once daily, 20 ml	14d	(1) (3) (6) (7)
Li et al. (2018)	Acute phase	50/50	51.90	52.30	Atorvastatin calcium, DHI	Atorvastatin calcium	Once daily, 20 ml	14d	(14)
Jin et al. (2019)	Acute phase	50/50	52.00	54.00	rt-PA, DHI	rt-PA	Once daily, 20 ml	14d	(1)
Chai et al. (2019)	Acute phase	42/42	64.08	63.17	Ozagrel Sodium, aspirin, DHI	Ozagrel Sodium, aspirin	Once daily, 40 ml	14d	(1) (4)
Chen et al. (2019)	Acute phase	74/74	54.69	52.61	CT, DHI	CT	Once daily, 20 ml	14d	(1)
Li and Tian., (2019)	Acute phase	42/42	63.49	63.58	Urinary Kallidinogenase, DHI	Urinary Kallidinogenase	Once daily, 20 ml	14d	(1) (14)
Zhu and Qi, (2020)	Acute phase	30/30	56.34	57.62	CT, DHI	CT	Once daily, 20 ml	14d	(1) (3)
Cao et al. (2019)	Acute phase	150/150	59.70	59.00	CT, DHI	CT	Once daily, 30 ml	14d	(13) (14)
Song and Yang, (2014)	Acute phase	65/65	65.23	65.89	CT, DHI	CT	Once daily, 30 ml	14d	(1) (3) (8) (9) (11) (13) (14)
Lv et al. (2018)	Acute phase	40/40	63.01	62.34	rt-PA, DHI	rt-PA	Once daily, 20 ml	14d	(3) (14)
Huo et al. (2020)	Acute phase	45/45	NR	NR	Deproteinized Calf Blood Injection, DHI	Deproteinized Calf Blood Injection	Once daily, 30 mg	14d	(1)
Cao and Mei., (2016)	Acute phase	34/33	54.50	58.90	CT, DHI	CT	Once daily, 30 ml	14d	(1) (14)
Ren et al. (2011)	Acute phase	34/34	62.20	63.10	CT, DHI	CT	Once daily, 40 ml	14d	(1)
Xue and Li., (2010)	NR	37/49	65.70	67.50	CT, DHI	CT	Once daily, 30 ml	14d	(5) (8) (10) (11) (14)

CG, control group; EG, experiment group (Danhong Injection group); NR, not reported; CT, conventional treatment.

Outcomes: (1) = NIHSS, (2) = Fugl-Meyer Assessment, (3) = Barthel index, (4) = ADL, (5) = IMT, (6) = cerebral blood flow, (7) = average cerebral blood flow rate, (8) = total cholesterol, (9) = triglycerides, (10) = HDL, (11) = LDL, (12) = Hcy, (13) = D-dimer, (14) = ADRs.

### Risk of bias

All RCTs reported methods of random sequence generation. Only three trials included (4%) blinded patients and caregivers (Liu and Huang 2010; Su et al., 2012; Liu, 2017). No details were identified in the domains of allocation concealment, selective outcome reporting, and other sources of bias; thus, we judged these items as unclear risk of bias (Supplementary Appendix 3, Table S1).

### National institute of health stroke scale analysis

**FIGURE 2:**
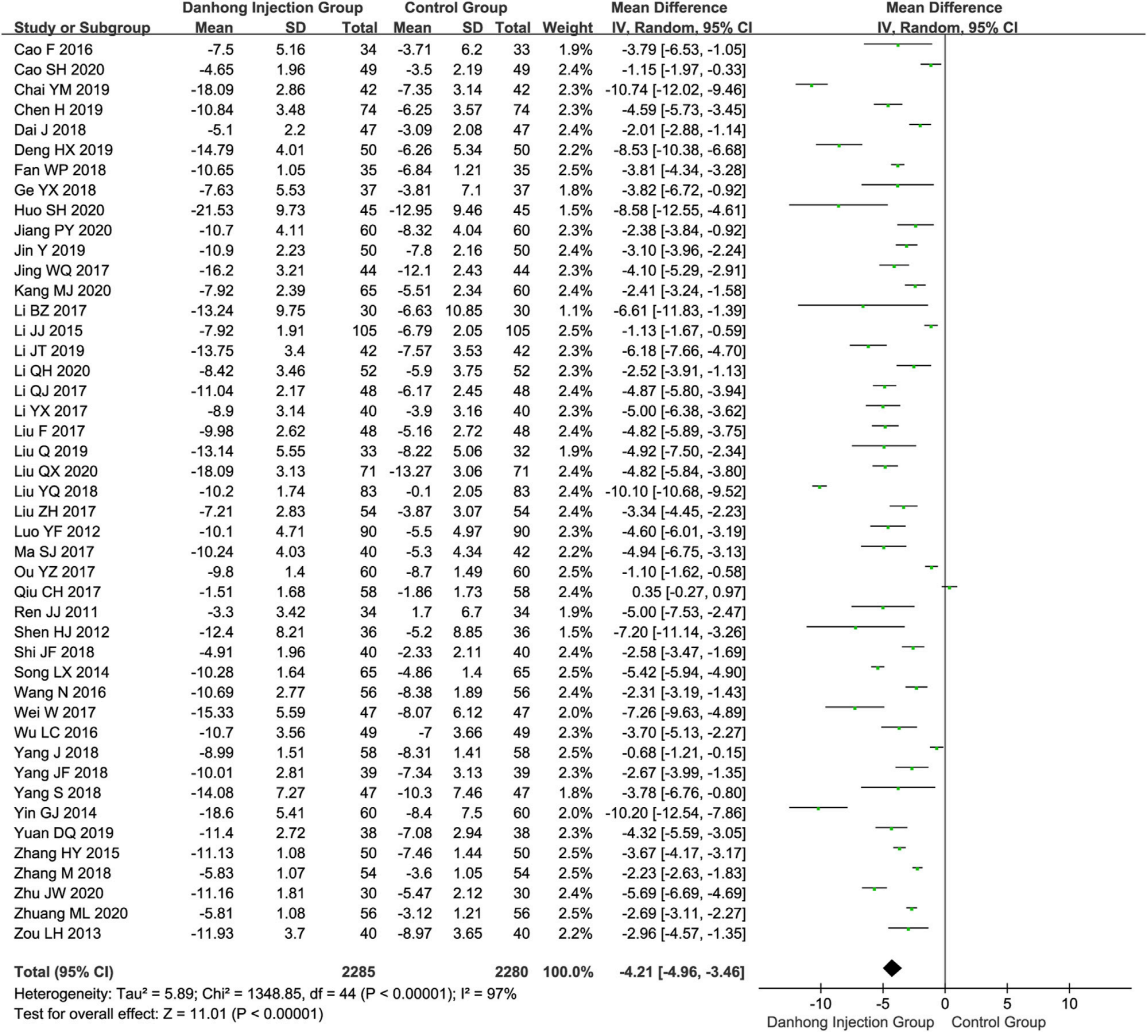
Meta-analysis of NIHSS between DHI + WM and WM in patients with ischemic stroke.

In total, 45 RCTs (Ren et al., 2011; Luo et al., 2012; Shen, 2012; Zou et al., 2013; Song and Yang 2014; Yin, 2014; Li, 2015; Zhang, 2015; Cao and Mei 2016; Wang et al., 2016; Wu and Bao, 2016; Li Q. et al., 2017; Liu F. et al., 2017; Li Y. et al., 2017; Liu Z. et al., 2017; Li, 2017; Ma, 2017; Ou and Liu 2017; Qiu et al., 2017; Wei et al., 2017; Yang and Liu, 2018a; Yang et al., 2018b; Dai, 2018; Fan et al., 2018; Ge, 2018; Liu, 2018; Shi, 2018; Yang, 2018; Zhang et al., 2018; Liu Q. et al., 2019; Chai et al., 2019; Chen et al., 2019; Deng, 2019; Jin et al., 2019; Li and Tian 2019; Yuan, 2019; Cao, 2020; Huo et al., 2020; Jiang et al., 2020; Jing and Lian, 2020; Kang et al., 2020; Liu and Jin, 2020; Zhu and Qi, 2020; Zhuang and Zhao, 2020) involving 4565 patients reported the NIHSS score. For Danhong injection combined with the Western medicine group, the NIHSS score significantly changed −10.50 (95% CI: −11.50 to −9.51), and the control group changed −6.22 (95% CI: −7.05 to −5.39). Meta-analysis showed that Dahong injection combined with Western medicine had a better effect in improving neurological impairment than Western medicine alone (NIHSS score MD −4.21, 95% CI −4.96 to −3.46) (Figure 2).

### Self-care ability analysis

**FIGURE 3:**
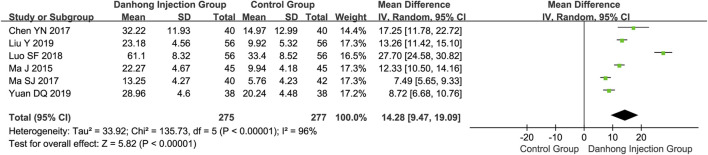
Meta-analysis of Fugl–Meyer Assessment between DHI + WM and WM in patients with ischemic stroke.

Six RCTs (Ma et al., 2015; Chen and Shi, 2017; Ma, 2017; Luo, 2018; Liu Y. et al., 2019; Yuan, 2019) involving 552 patients showed that Danhong injection combined with Western medicine could significantly improve the Fugl–Meyer assessment than Western medicine alone (MD 14.28, 95% CI 9.47–19.09) (Figure 3). Compared with Western medicine alone, Danhong injection plus Western medicine suggested a significant improvement in the Barthel index (14 trials with 1,270 patients; MD 8.71, 95% CI 3.68–13.74) and the ADL score (12 trials with 1,114 patients; MD 14.48, 95% CI 9.04–19.92) (Supplementary Figures S1, S2, Appendix 2).

### Hemodynamic outcomes analysis

Meta-analysis showed that compared with Western medicine alone, the combination therapy of Danhong injection and Western medicine could significantly lower the IMT (three trials with 284 patients; MD −0.23 mm, 95% CI −0.26 to −0.20), increase the cerebral blood flow (two trials with 200 patients; MD 1.16 ml/s, 95% CI 0.64–1.68), and average cerebral blood flow rate (five trials with 508 patients; MD 3.05 cm/s, 95% CI 1.61–4.50) (Supplementary Figures S3–S5, Appendix 2).

### Blood lipid analysis

**FIGURE 4:**
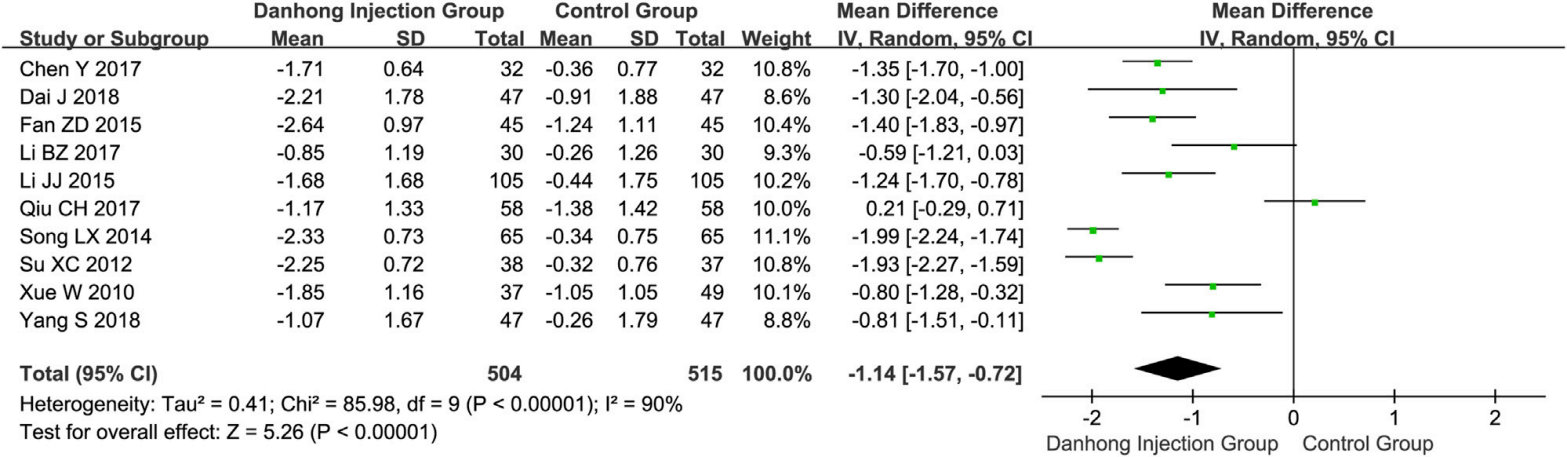
Meta-analysis of total cholesterol between DHI + WM and WM in patients with ischemic stroke.

The combination therapy of Danhong injection and Western medicine could significantly lower total cholesterol (10 trials with 1019 patients; MD −1.14 mmol/L, 95% CI −1.57 to −0.72) (Figure 4), triglycerides (eight trials with 869 patients; MD −1.00 mmol/L, 95% CI −1.69 to −0.31), and LDL (five trials with 441 patients; MD −0.91 mmol/L, 95% CI −1.33 to −0.49), and increase the level of HDL (three trials with 236 patients; MD 0.31 mmol/L, 95% CI 0.22–0.40) (Supplementary Figures S6–S8, Appendix 2).

### Hcy analysis

**FIGURE 5:**
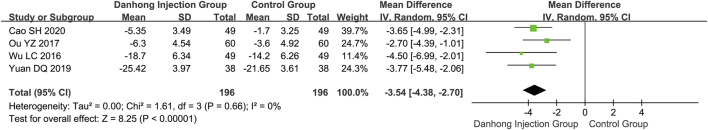
Meta-analysis of Hcy between DHI + WM and WM in patients with ischemic stroke.

Four RCTs (Wu and Bao, 2016; Ou and Liu 2017; Yuan, 2019; Cao, 2020) with a total of 392 individuals reported the data of serum Hcy level. The results revealed a significant lowering effect on Hcy in the Danhong injection combination therapy (MD −3.54 μmol/L, 95% CI −4.38 to −2.07) (Figure 5).

### D-dimer analysis

Meta-analysis of five trials with a total of 612 patients showed no statistically significant difference in D-dimer (MD −0.12 mg/L, 95% CI −0.61–0.37, *p* = 0.64) between treatment groups (Supplementary Figure S9, Appendix 2).

### Subgroup analysis

Subgroup analysis by mean age showed differential effects in average cerebral blood flow rate (<60 years old: MD 0.74 cm/s, 95% CI 0.29–1.19; ≥60 years old: MD 4.09 cm/s, 95% CI 2.02–6.16; interaction *p* = 0.002). The analysis by length of treatment showed differential effects in ADL (≤2 weeks: MD 15.08, 95% CI 9.11–21.05; >2 weeks: MD 8.18, 95% CI 6.21–10.15; interaction *p* = 0.03). Subgroup analysis by the type of baseline Western medicines showed differential effects (conventional therapy: MD −3.79, 95% CI −4.83 to −2.76; intravenous thrombolysis: MD −2.19, 95% CI −3.52 to −0.86; anti-platelet aggregation: MD −3.79, 95% CI −5.06 to −2.51; dilation of blood vessels: MD −4.23, 95% CI −5.85 to −2.62; statins: MD −2.87, 95% CI −4.07 to −1.67; neuroprotective agents: MD −5.88, 95% CI −8.90 to −2.87; other therapies: MD −10.20, 95% CI −12.54 to −7.86; interaction *p* < 0.00001).

As for the Fugl-Meyer assessment, the Barthel index, total cholesterol, triglycerides, LDL, Hcy, and D-dimer, subgroup analyses by length of treatment, mean age of patients, and types of baseline Western medicine treatment showed no statistically differential effects.

### Safety

Of the 67 included trials, 16 studies (Liu and Huang 2010; Shen, 2012; Wang, 2016; Li Q. et al., 2017; Li Y. et al., 2017; Chen and Shi, 2017; Wei et al., 2017; Zhang, 2017; Yang ad Liu 2018a; Ge, 2018; Li et al., 2018; Liu, 2018; Lv et al., 2018; Zhang et al., 2018; Li and Tian, 2019; Zhuang and Zhao, 2020) reported at least one case of ADRs, 12 studies (Xue and Li, 2010; Su et al., 2012; Song and yang, 2014; Cao and Mei, 2016; Wu and Bao, 2016; Luo, 2018; Cao et al., 2019; Cheng, 2019; Zhang, 2019; Cao, 2020; Jiang et al., 2020; Kang et al., 2020) reported no ADRs/ADEs during the study, and 39 RCTs did not mention the information of ADRs/ADEs.

The main ADRs that occurred during the trials were gastrointestinal reactions (4.48% vs. 4.76% in Danhong injection plus Western medicine and Western medicine alone group, respectively, *p* = 0.76), dizziness (3.69% vs. 2.74% in each group, *p* = 0.61), and skin rash (8.53% vs. 7.31% in each group, *p* = 0.56). ADRs that occurred during the trials in either group are shown in Supplementary Appendix 3, Table S2.

### Assessment of publication bias

The funnel plots for NIHSS, Barthel index, and ADL were near asymmetric, and no important publication bias was detected by Egger’s test (Egger’s test *p* = 0.055, *p* = 0.342 and *p* = 0.998, respectively). Egger’s test of total cholesterol suggested potential publication bias (Egger’s test *p* = 0.019). The sensitivity analysis by the trim-and-fill method showed that the results were robust despite the potential bias (trim-and-fill method adjusted MD −1.14 mmol/L, 95% CI −1.57 to −0.72) (Supplementary Figures S10, S11, Appendix 2).

## Discussion

### Findings and interpretations

In this systematic review and meta-analysis of 67 randomized controlled trials, we found that adding Danhong injection to Western medicine could significantly improve neurological function (e.g., improving NIHSS, the Fugl–Meyer assessment), self-care ability (e.g., improving the Barthel index and ADL scores), hemodynamic outcomes (cerebral blood flow, average cerebral blood flow rate), blood lipids, and Hcy in patients with ischemic stroke. Furthermore, patients of different ages and receiving different baseline Western medicines may have different benefits in hemodynamic status and neurological function.

NIHSS is a stroke-specific quantitative scale with excellent reliability and validity for ischemic stroke outcomes and measures stroke-related neurological deficits such as the level of consciousness, language function, visual field, eye movement sensory function, and coordination (Muir et al., 1996; Kasner et al., 1999; Boone et al., 2012). In our study, we found that NIHSS scores were significantly decreased in patients receiving Danhong injection plus Western medicine. The minimal clinically important difference (MCID) of the NIHSS score in stroke patients was considered an increase or decrease of two points, that is, a change in the NIHSS score of more than two points suggests clinical importance of the differences (Chang and Xu., 2011; Neurology Chapter of China Association of Chinese Medicine et al., 2020; Zeitlberger et al., 2021). The change of the NIHSS score in our study was 4.21 points between the experiment group and the control group, which suggested that Danhong injection added to Western medicine would be more beneficial in reducing the severity of ischemic stroke and improving neurological status than Western medicine alone for ischemic stroke patients. Overactivation of inflammatory factors and aggregation of platelets cause thrombosis and can lead to vascular occlusion, resulting in cerebrovascular ischemia, and then contribute to neurological deficit and physical dysfunction (Franks et al., 2010; Xu et al., 2016; Komurcu et al., 2020). Since Danhong injection could inhibit platelet aggregation and boost the activity of fibrinogen dissolved, the add-on effects of Danhong injection and Western medicine play a key role in improving NIHSS.

The Fugl–Meyer assessment is considered one of the most comprehensive and evaluative measures for assessment of recovery in poststroke patients (Gladstone et al., 2002). As approximately 85% of patients with stroke present with arm weakness (Dawson et al., 2016), our study found that the scores in the Fugl–Meyer assessment were significantly increased in patients treated by the integrated treatment with Danhong injection and Western medicine, which suggests that Danhong injection plus Western medicine have the potential of enhancing limb function in stroke recovery.

PPAR-α, a transcription factor that regulates diverse aspects of lipid metabolism, plays a key role in the regulation of hepatic lipid metabolism (Kersten et al., 1999; Li and Glass, 2004). Our study showed that the combination of Danhong injection and Western medicine had a noteworthy effect on reducing blood lipids (e.g., total cholesterol, triglycerides, and LDL). Our study also showed that Danhong injection plus Western medicine had a marked impact on increasing cerebral blood flow and average cerebral blood flow rate, which can be explained with the fact that Danhong injection could improve hemodynamic indices such as high shear viscosity, low shear viscosity, hematocrit, and platelet aggregation rate (Jiang and Lian, 2015).

Our study found the most common ADRs in these reported RCTs were gastrointestinal reactions including nausea, flatulence, and vomiting, which are a consequence of the drug’s normal pharmacological effects (Li et al., 2015). About 58.2% of RCTs did not mention the information of ADRs in this study; thus, we were unable to make a conclusion on safety so far. More credible evidence is warranted to confirm the safety of the add-on effect of this drug in the future.

### Comparison with other studies

Three previous systematic reviews and meta-analyses have assessed the effect of Danhong injection on the treatment of ischemic stroke (Wang et al., 2017; Liu et al., 2018; Liu et al., 2019b). In terms of efficacy, all these three studies compared Danhong injection with other Chinese botanical drug injections such as Shuxuening injection, Yinxingdamo injection, and so on. Instead of making a claim of comparative effectiveness between traditional Chinese medicines, we aimed to address whether the addition of traditional Chinese medicine to routinely used medications would improve the effects on ischemic stroke, which was a question that needed strong evidence for conclusion. As it turned out, our study has provided a reliable assessment of the effects.

### Strengths and limitations

Our study has several strengths. First, we used rigorous methods to search, screen, and collect data from eligible RCTs. Second, we included several newly published RCTs to summarize the efficacy and safety of Danhong injection in patients with ischemic stroke, aiming to provide a more comprehensive, up-to-date, and new level of clinical evidence. Third, we included a large number of RCTs and used both patients who reported important outcomes (e.g., NIHSS, Fugl-Meyer Assessment scores) and other objective outcomes (e.g., blood lipids, hemodynamic outcomes) to comprehensively assess the efficacy and safety of Danhong injection in patients with ischemic stroke. Fourth, we conducted three prespecified subgroup analyses to explore sources of heterogeneity.

However, our study also has some limitations. The included RCTs may be at a risk of high risk of bias. As a result, the findings may be susceptible to the bias. Additional well-designed and rigorous trials with long-term outcomes may be needed to further consolidate our findings.

## Conclusion

In summary, the available evidence suggests that Danhong injection plus Western medicine may improve the effects of neurological function, self-care ability, hemodynamic status, blood lipids, and Hcy for patients with ischemic stroke. However, given that most included trials presented with unclear risk of bias, current evidence is inconclusive and should be explained with caution. Therefore, more carefully designed and conducted trials are needed to confirm the add-on effects of Danhong injection.

## Data Availability

The original contributions presented in the study are included in the article/Supplementary Material; further inquiries can be directed to the corresponding authors.

## Abbreviations

ADEs, adverse drug events; ADL, activities of daily living; ADRs, adverse drug reactions; CIs, confidence intervals; CNKI, China national knowledge infrastructure database; Hcy, homocysteine; HDL, high-density lipoprotein; IMT, intima-media thickness; LDL, low-density lipoprotein; MCID, minimal clinically important difference; MD, mean difference; NIHSS, national institutes of health stroke scale score; NR, not reported; PRISMA, preferred reporting items for systematic reviews and meta-analyses; RCTs, randomized controlled trials
