# Supplementary material for: The add-on effects of Danhong injection among patients with ischemic stroke receiving Western medicines: A systematic review and meta-analysis
Source: Front Pharmacol. 2022 Aug 23;13:937369. doi: 10.3389/fphar.2022.937369 (PMC9445550; doi:10.3389/fphar.2022.937369)
Supplement: Supplementary file 1 [file DataSheet4.docx]

**Summary table of all of the papers cited**

| Study | Formulation | Source | Species, concentration | Quality control reported? (Y/N) | Chemical analysis reported? (Y/N) |
| --- | --- | --- | --- | --- | --- |
| Cao 2020 | Danhong injection | [Shandong Danhong Pharmaceutical Co., Ltd, Z20026866] | - Salvia miltiorrhiza Bunge., 0.75g/ ml - Carthamus tinctorius L.,0.25g/ ml | N | N |
| Cheng 2019 | Danhong injection | [Heze Buchang Pharmaceutical Co., Ltd, Z20026866] | - Salvia miltiorrhiza Bunge., 0.75g/ ml - Carthamus tinctorius L.,0.25g/ ml | N | N |
| Zhang 2019 | Danhong injection | [Jinan Buchang Pharmaceutical Co., Ltd, Sinopharm Z20026866] | - Salvia miltiorrhiza Bunge., 0.75g/ ml - Carthamus tinctorius L.,0.25g/ ml | N | N |
| Deng 2019 | Danhong injection | [Shandong Danhong Pharmaceutical Co., Ltd, Sinopharm Z20026866] | - Salvia miltiorrhiza Bunge., 0.75g/ ml - Carthamus tinctorius L.,0.25g/ ml | N | N |
| Li 2017 | Danhong injection | [Xianyang Buchang Pharmaceutical Co., Ltd, Sinopharm Z20026866] | - Salvia miltiorrhiza Bunge., 0.75g/ ml - Carthamus tinctorius L.,0.25g/ ml | N | N |
| Li 2017 | Danhong injection | [Shandong Danhong Pharmaceutical Co., Ltd, Z20026866] | - Salvia miltiorrhiza Bunge., 0.75g/ ml - Carthamus tinctorius L.,0.25g/ ml | N | N |
| Wang 2016 | Danhong injection | [Buchang Pharmaceutical Co., Ltd, Z20026866] | - Salvia miltiorrhiza Bunge., 0.75g/ ml - Carthamus tinctorius L.,0.25g/ ml | N | N |
| Zhang 2015 | Danhong injection | [Not reported] | - Salvia miltiorrhiza Bunge., Not applicable - Carthamus tinctorius L., Not applicable | N | N |
| Yin 2014 | Danhong injection | [Heze Buchang Pharmaceutical Co., Ltd, Z20026866] | - Salvia miltiorrhiza Bunge., 0.75g/ ml - Carthamus tinctorius L.,0.25g/ ml | N | N |
| Shen 2012 | Danhong injection | [Heze Buchang Pharmaceutical Co., Ltd, Z20026866] | - Salvia miltiorrhiza Bunge., 0.75g/ ml - Carthamus tinctorius L.,0.25g/ ml | N | N |
| Zhuang 2020 | Danhong injection | [Heze Buchang Pharmaceutical Co., Ltd, Z20026866] | - Salvia miltiorrhiza Bunge., 0.75g/ ml - Carthamus tinctorius L.,0.25g/ ml | N | N |
| Liu 2018 | Danhong injection | [Shandong Danhong Pharmaceutical Co., Ltd, Z20026866] | - Salvia miltiorrhiza Bunge., 0.75g/ ml - Carthamus tinctorius L.,0.25g/ ml | N | N |
| Shi 2018 | Danhong injection | [Not reported] | - Salvia miltiorrhiza Bunge., Not applicable - Carthamus tinctorius L., Not applicable | N | N |
| Yang 2018 | Danhong injection | [Jinan Buchang Pharmaceutical Co., Ltd, Z20026866] | - Salvia miltiorrhiza Bunge., 0.75g/ ml - Carthamus tinctorius L.,0.25g/ ml | N | N |
| Ma 2017 | Danhong injection | [Danhong Pharmaceutical Co., Ltd, Z20026866] | - Salvia miltiorrhiza Bunge., 0.75g/ ml - Carthamus tinctorius L.,0.25g/ ml | N | N |
| Liu 2010 | Danhong injection | [Not reported] | - Salvia miltiorrhiza Bunge., Not applicable - Carthamus tinctorius L., Not applicable | N | N |
| Chen 2017 | Danhong injection | [Shandong Danhong Pharmaceutical Co., Ltd, Z20026866] | - Salvia miltiorrhiza Bunge., 0.75g/ ml - Carthamus tinctorius L.,0.25g/ ml | N | N |
| Zhang 2018 | Danhong injection | [Shandong Buchang Pharmaceutical Co., Ltd, Sinopharm Z20031672] | - Salvia miltiorrhiza Bunge., Not applicable - Carthamus tinctorius L., Not applicable | N | N |
| Zhang 2017 | Danhong injection | [Shandong Buchang Pharmaceutical Co., Ltd, Z20026866] | - Salvia miltiorrhiza Bunge., 0.75g/ ml - Carthamus tinctorius L.,0.25g/ ml | N | N |
| Pen 2017 | Danhong injection | [Jinan Buchang Pharmaceutical Co., Ltd., Z20026866] | - Salvia miltiorrhiza Bunge., 0.75g/ ml - Carthamus tinctorius L.,0.25g/ ml | N | N |
| Fan 2018 | Danhong injection | [Not reported] | - Salvia miltiorrhiza Bunge., Not applicable - Carthamus tinctorius L., Not applicable | N | N |
| Li 2017 | Danhong injection | [Not reported] | - Salvia miltiorrhiza Bunge., Not applicable - Carthamus tinctorius L., Not applicable | N | N |
| Jiang 2020 | Danhong injection | [Shandong Danhong Pharmaceutical Co., Ltd, Z20026866] | - Salvia miltiorrhiza Bunge., 0.75g/ ml - Carthamus tinctorius L.,0.25g/ ml | N | N |
| Liu 2019 | Danhong injection | [Shandong Danhong Pharmaceutical Co., Ltd, Z20026866] | - Salvia miltiorrhiza Bunge., 0.75g/ ml - Carthamus tinctorius L.,0.25g/ ml | N | N |
| Liu 2017 | Danhong injection | [Heze Buchang Pharmaceutical Co., Ltd, Z20026866] | - Salvia miltiorrhiza Bunge., 0.75g/ ml - Carthamus tinctorius L.,0.25g/ ml | N | N |
| Ge 2018 | Danhong injection | [Shandong Danhong Pharmaceutical Co., Ltd, Z20026866] | - Salvia miltiorrhiza Bunge., 0.75g/ ml - Carthamus tinctorius L.,0.25g/ ml | N | N |
| Yun 2017 | Danhong injection | [Shandong Danhong Pharmaceutical Co., Ltd., Z20026866] | - Salvia miltiorrhiza Bunge., 0.75g/ ml - Carthamus tinctorius L.,0.25g/ ml | N | N |
| Liu 2019 | Danhong injection | [Heze Buchang Pharmaceutical Co., Ltd, Z20026866] | - Salvia miltiorrhiza Bunge., 0.75g/ ml - Carthamus tinctorius L.,0.25g/ ml | N | N |
| Dai 2018 | Danhong injection | [Heze Buchang Pharmaceutical Co., Ltd, Z20026866] | - Salvia miltiorrhiza Bunge., 0.75g/ ml - Carthamus tinctorius L.,0.25g/ ml | N | N |
| Yang 2018 | Danhong injection | [Shandong Danhong Pharmaceutical Co., Ltd., Z20026866] | - Salvia miltiorrhiza Bunge., 0.75g/ ml - Carthamus tinctorius L.,0.25g/ ml | N | N |
| Li 2015 | Danhong injection | [Not reported] | - Salvia miltiorrhiza Bunge., Not applicable - Carthamus tinctorius L., Not applicable | N | N |
| Li 2020 | Danhong injection | [Not reported] | - Salvia miltiorrhiza Bunge., Not applicable - Carthamus tinctorius L., Not applicable | N | N |
| Liu 2017 | Danhong injection | [Heze Buchang Pharmaceutical Co., Ltd, Z20026866] | - Salvia miltiorrhiza Bunge., 0.75g/ ml - Carthamus tinctorius L.,0.25g/ ml | N | N |
| Luo 2018 | Danhong injection | [Sichuan Shenghe Pharmaceutical Co., Ltd, Z51021303] | - Salvia miltiorrhiza Bunge., Not applicable - Carthamus tinctorius L., Not applicable | N | N |
| Kang 2020 | Danhong injection | [Shandong Danhong Pharmaceutical Co., Ltd., Z20026866] | - Salvia miltiorrhiza Bunge., 0.75g/ ml - Carthamus tinctorius L.,0.25g/ ml | N | N |
| Yang 2018 | Danhong injection | [Shandong Danhong Pharmaceutical Co., Ltd., Z20026866] | - Salvia miltiorrhiza Bunge., 0.75g/ ml - Carthamus tinctorius L.,0.25g/ ml | N | N |
| Liu 2020 | Danhong injection | [Shandong Danhong Pharmaceutical Co., Ltd, Z20026866] | - Salvia miltiorrhiza Bunge., 0.75g/ ml - Carthamus tinctorius L.,0.25g/ ml | N | N |
| Wang 2016 | Danhong injection | [Shandong Danhong Pharmaceutical Co., Ltd, Z20026866] | - Salvia miltiorrhiza Bunge., 0.75g/ ml - Carthamus tinctorius L.,0.25g/ ml | N | N |
| Yuan 2019 | Danhong injection | [Shandong Danhong Pharmaceutical Co., Ltd, Z20026866] | - Salvia miltiorrhiza Bunge., 0.75g/ ml - Carthamus tinctorius L.,0.25g/ ml | N | N |
| Su 2012 | Danhong injection | [Jinan Buchang Pharmaceutical Co., Ltd., Z20026866] | - Salvia miltiorrhiza Bunge., 0.75g/ ml - Carthamus tinctorius L.,0.25g/ ml | N | N |
| Luo 2012 | Danhong injection | [Heze Buchang Pharmaceutical Co., Ltd, Z20026866] | - Salvia miltiorrhiza Bunge., 0.75g/ ml - Carthamus tinctorius L.,0.25g/ ml | N | N |
| Zou 2013 | Danhong injection | [Heze Buchang Pharmaceutical Co., Ltd, Z20026866] | - Salvia miltiorrhiza Bunge., 0.75g/ ml - Carthamus tinctorius L.,0.25g/ ml | N | N |
| Liang 2014 | Danhong injection | [Xi’an Buchang Pharmaceutical Co., Ltd, Z20026866] | - Salvia miltiorrhiza Bunge., 0.75g/ ml - Carthamus tinctorius L.,0.25g/ ml | N | N |
| Ma 2015 | Danhong injection | [Heze Buchang Pharmaceutical Co., Ltd, Z20026866] | - Salvia miltiorrhiza Bunge., 0.75g/ ml - Carthamus tinctorius L.,0.25g/ ml | N | N |
| Fan 2015 | Danhong injection | [Heze Buchang Pharmaceutical Co., Ltd, Z20026866] | - Salvia miltiorrhiza Bunge., 0.75g/ ml - Carthamus tinctorius L.,0.25g/ ml | N | N |
| Zeng 2016 | Danhong injection | [Shandong Danhong Pharmaceutical Co., Ltd, Z20026866] | - Salvia miltiorrhiza Bunge., 0.75g/ ml - Carthamus tinctorius L.,0.25g/ ml | N | N |
| Feng 2016 | Danhong injection | [Heze Buchang Pharmaceutical Co., Ltd, Z20026866] | - Salvia miltiorrhiza Bunge., 0.75g/ ml - Carthamus tinctorius L.,0.25g/ ml | N | N |
| Ou 2017 | Danhong injection | [Heze Buchang Pharmaceutical Co., Ltd, Z20026866] | - Salvia miltiorrhiza Bunge., 0.75g/ ml - Carthamus tinctorius L.,0.25g/ ml | N | N |
| Wu 2016 | Danhong injection | [Heze Buchang Pharmaceutical Co., Ltd., Z20026866] | - Salvia miltiorrhiza Bunge., 0.75g/ ml - Carthamus tinctorius L.,0.25g/ ml | N | N |
| Chen 2017 | Danhong injection | [Heze Buchang Pharmaceutical Co., Ltd, Z20026866] | - Salvia miltiorrhiza Bunge., 0.75g/ ml - Carthamus tinctorius L.,0.25g/ ml | N | N |
| Qiu 2017 | Danhong injection | [Shandong Buchang Pharmaceutical Co., Ltd, Z20026866] | - Salvia miltiorrhiza Bunge., 0.75g/ ml - Carthamus tinctorius L.,0.25g/ ml | N | N |
| Liu 2017 | Danhong injection | [Shandong Danhong Pharmaceutical Co., Ltd, Z20026866] | - Salvia miltiorrhiza Bunge., 0.75g/ ml - Carthamus tinctorius L.,0.25g/ ml | N | N |
| Wei 2017 | Danhong injection | [Heze Buchang Pharmaceutical Co., Ltd, Z20026866] | - Salvia miltiorrhiza Bunge., 0.75g/ ml - Carthamus tinctorius L.,0.25g/ ml | N | N |
| Jing 2020 | Danhong injection | [Shandong Danhong Pharmaceutical Co., Ltd, Z20026866] | - Salvia miltiorrhiza Bunge., 0.75g/ ml - Carthamus tinctorius L.,0.25g/ ml | N | N |
| Li 2018 | Danhong injection | [Heze Buchang Pharmaceutical Co., Ltd, Z20026866] | - Salvia miltiorrhiza Bunge., 0.75g/ ml - Carthamus tinctorius L.,0.25g/ ml | N | N |
| Jin 2019 | Danhong injection | [Heze Buchang Pharmaceutical Co., Ltd, Z20026866] | - Salvia miltiorrhiza Bunge., 0.75g/ ml - Carthamus tinctorius L.,0.25g/ ml | N | N |
| Chai 2019 | Danhong injection | [Shandong Danhong Pharmaceutical Co., Ltd, Z20026866] | - Salvia miltiorrhiza Bunge., 0.75g/ ml - Carthamus tinctorius L.,0.25g/ ml | N | N |
| Chen 2019 | Danhong injection | [Heze Buchang Pharmaceutical Co., Ltd, Z20026866] | - Salvia miltiorrhiza Bunge., 0.75g/ ml - Carthamus tinctorius L.,0.25g/ ml | N | N |
| Li 2019 | Danhong injection | [Heze Buchang Pharmaceutical Co., Ltd, Z20026866] | - Salvia miltiorrhiza Bunge., 0.75g/ ml - Carthamus tinctorius L.,0.25g/ ml | N | N |
| Zhu 2020 | Danhong injection | [Heze Buchang Pharmaceutical Co., Ltd, Z20026866] | - Salvia miltiorrhiza Bunge., 0.75g/ ml - Carthamus tinctorius L.,0.25g/ ml | N | N |
| Cao 2019 | Danhong injection | [Shandong Danhong Pharmaceutical Co., Ltd, Z20026866] | - Salvia miltiorrhiza Bunge., 0.75g/ ml - Carthamus tinctorius L.,0.25g/ ml | N | N |
| Song 2014 | Danhong injection | [Not reported] | - Salvia miltiorrhiza Bunge., Not applicable - Carthamus tinctorius L., Not applicable | N | N |
| Lv 2018 | Danhong injection | [Shandong Danghong Pharmaceutical Co., Ltd, Z20026866] | - Salvia miltiorrhiza Bunge., 0.75g/ ml - Carthamus tinctorius L.,0.25g/ ml | N | N |
| Huo 2020 | Danhong injection | [Shandong Danghong Pharmaceutical Co., Ltd, Z20026866] | - Salvia miltiorrhiza Bunge., 0.75g/ ml - Carthamus tinctorius L.,0.25g/ ml | N | N |
| Cao 2016 | Danhong injection | [Shandong Danghong Pharmaceutical Co., Ltd, Z20026866] | - Salvia miltiorrhiza Bunge., 0.75g/ ml - Carthamus tinctorius L.,0.25g/ ml | N | N |
| Ren 2011 | Danhong injection | [Jinan Buchang Pharmaceutical Co., Ltd., Z20026866] | - Salvia miltiorrhiza Bunge., 0.75g/ ml - Carthamus tinctorius L.,0.25g/ ml | N | N |
| Xue 2010 | Danhong injection | [Not reported] | - Salvia miltiorrhiza Bunge., Not applicable - Carthamus tinctorius L., Not applicable | N | N |
