# Supplementary material for: The add-on effects of Danhong injection among patients with ischemic stroke receiving Western medicines: A systematic review and meta-analysis
Source: Front Pharmacol. 2022 Aug 23;13:937369. doi: 10.3389/fphar.2022.937369 (PMC9445550; doi:10.3389/fphar.2022.937369)
Supplement: Supplementary file 2 [file DataSheet3.docx]

**Appendix 3** Supplementary tables

**Table S1.** Risk of bias of randomized controlled trials

| **Author (year)** | **Randomization sequence generation** | **Allocation concealment** | **Blinding of participants and personnel** | **Blinding of outcome assessment** | **Incomplete outcome data** | **Selective outcome reporting** | **Other sources of bias*** |
| --- | --- | --- | --- | --- | --- | --- | --- |
| Cao 2020 | Low | Unclear | Unclear | Unclear | Low | Unclear | Unclear |
| Cheng 2019 | Low | Unclear | Unclear | Unclear | Low | Unclear | Unclear |
| Zhang 2019 | Low | Unclear | Unclear | Unclear | Low | Unclear | Unclear |
| Deng 2019 | Low | Unclear | Unclear | Unclear | Low | Unclear | Unclear |
| Li 2017 | Low | Unclear | Unclear | Unclear | Low | Unclear | Unclear |
| Li 2017 | Low | Unclear | Unclear | Unclear | Low | Unclear | Unclear |
| Wang 2016 | Low | Unclear | Unclear | Unclear | Low | Unclear | Unclear |
| Zhang 2015 | Low | Unclear | Unclear | Unclear | Low | Unclear | Unclear |
| Yin 2014 | Low | Unclear | Unclear | Unclear | Low | Unclear | Unclear |
| Shen 2012 | Low | Unclear | Unclear | Unclear | Low | Unclear | Unclear |
| Zhuang 2020 | Low | Unclear | Unclear | Unclear | Low | Unclear | Unclear |
| Liu 2018 | Low | Unclear | Unclear | Unclear | Low | Unclear | Unclear |
| Shi 2018 | Low | Unclear | Unclear | Unclear | Low | Unclear | Unclear |
| Yang 2018 | Low | Unclear | Unclear | Unclear | Low | Unclear | Unclear |
| Ma 2017 | Low | Unclear | Unclear | Unclear | Low | Unclear | Unclear |
| Liu 2010 | Low | Unclear | Low | Unclear | Low | Unclear | Unclear |
| Chen 2017 | Low | Unclear | Unclear | Unclear | Low | Unclear | Unclear |
| Zhang 2018 | Low | Unclear | Unclear | Unclear | Low | Unclear | Unclear |
| Zhang 2017 | Low | Unclear | Unclear | Unclear | Low | Unclear | Unclear |
| Pen 2017 | Low | Unclear | Unclear | Unclear | Low | Unclear | Unclear |
| Fan 2018 | Low | Unclear | Unclear | Unclear | Low | Unclear | Unclear |
| Li 2017 | Low | Unclear | Unclear | Unclear | Low | Unclear | Unclear |
| Jiang 2020 | Low | Unclear | Unclear | Unclear | Low | Unclear | Unclear |
| Liu 2019 | Low | Unclear | Unclear | Unclear | Low | Unclear | Unclear |
| Liu 2017 | Low | Unclear | Low | Unclear | Low | Unclear | Unclear |
| Ge 2018 | Low | Unclear | Unclear | Unclear | Low | Unclear | Unclear |
| Yun 2017 | Low | Unclear | Unclear | Unclear | Low | Unclear | Unclear |
| Liu 2019 | Low | Unclear | Unclear | Unclear | Low | Unclear | Unclear |
| Dai 2018 | Low | Unclear | Unclear | Unclear | Low | Unclear | Unclear |
| Yang 2018 | Low | Unclear | Unclear | Unclear | Low | Unclear | Unclear |
| Li 2015 | Low | Unclear | Unclear | Unclear | Low | Unclear | Unclear |
| Li 2020 | Low | Unclear | Unclear | Unclear | Low | Unclear | Unclear |
| Liu 2017 | Low | Unclear | Unclear | Unclear | Low | Unclear | Unclear |
| Luo 2018 | Low | Unclear | Unclear | Unclear | Low | Unclear | Unclear |
| Kang 2020 | Low | Unclear | Unclear | Unclear | Low | Unclear | Unclear |
| Yang 2018 | Low | Unclear | Unclear | Unclear | Low | Unclear | Unclear |
| Liu 2020 | Low | Unclear | Unclear | Unclear | Low | Unclear | Unclear |
| Wang 2016 | Low | Unclear | Unclear | Unclear | Low | Unclear | Unclear |
| Yuan 2019 | Low | Unclear | Unclear | Unclear | Low | Unclear | Unclear |
| Su 2012 | Low | Unclear | Low | Unclear | Low | Unclear | Unclear |
| Luo 2012 | Low | Unclear | Unclear | Unclear | Low | Unclear | Unclear |
| Zou 2013 | Low | Unclear | Unclear | Unclear | Low | Unclear | Unclear |
| Liang 2014 | Low | Unclear | Unclear | Unclear | Low | Unclear | Unclear |
| Ma 2015 | Low | Unclear | Unclear | Unclear | Low | Unclear | Unclear |
| Fan 2015 | Low | Unclear | Unclear | Unclear | Low | Unclear | Unclear |
| Zeng 2016 | Low | Unclear | Unclear | Unclear | Low | Unclear | Unclear |
| Feng 2016 | Low | Unclear | Unclear | Unclear | Low | Unclear | Unclear |
| Ou 2017 | Low | Unclear | Unclear | Unclear | Low | Unclear | Unclear |
| Wu 2016 | Low | Unclear | Unclear | Unclear | Low | Unclear | Unclear |
| Chen 2017 | Low | Unclear | Unclear | Unclear | Low | Unclear | Unclear |
| Qiu 2017 | Low | Unclear | Unclear | Unclear | Low | Unclear | Unclear |
| Liu 2017 | Low | Unclear | Unclear | Unclear | Low | Unclear | Unclear |
| Wei 2017 | Low | Unclear | Unclear | Unclear | Low | Unclear | Unclear |
| Jing 2020 | Low | Unclear | Unclear | Unclear | Low | Unclear | Unclear |
| Li 2018 | Low | Unclear | Unclear | Unclear | Low | Unclear | Unclear |
| Jin 2019 | Low | Unclear | Unclear | Unclear | Low | Unclear | Unclear |
| Chai 2019 | Low | Unclear | Unclear | Unclear | Low | Unclear | Unclear |
| Chen 2019 | Low | Unclear | Unclear | Unclear | Low | Unclear | Unclear |
| Li 2019 | Low | Unclear | Unclear | Unclear | Low | Unclear | Unclear |
| Zhu 2020 | Low | Unclear | Unclear | Unclear | Low | Unclear | Unclear |
| Cao 2019 | Low | Unclear | Unclear | Unclear | Low | Unclear | Unclear |
| Song 2014 | Low | Unclear | Unclear | Unclear | Low | Unclear | Unclear |
| Lv 2018 | Low | Unclear | Unclear | Unclear | Low | Unclear | Unclear |
| Huo 2020 | Low | Unclear | Unclear | Unclear | Low | Unclear | Unclear |
| Cao 2016 | Low | Unclear | Unclear | Unclear | Low | Unclear | Unclear |
| Ren 2011 | Low | Unclear | Unclear | Unclear | Low | Unclear | Unclear |
| Xue 2010 | Low | Unclear | Unclear | Unclear | Low | Unclear | Unclear |

*other sources of bias such as when assessing the same scale, the different qualification of different medical practitioners leads to bias in the assessment of the results.

**Table S2.** Summary of ADRs in DHI Group and Control Group

| **ADRs** | **Danhong Injection Group (n)** | **Control Group (n)** |
| --- | --- | --- |
| Gastrointestinal reactions | 16 | 17 |
| Dizzy | 11 | 17 |
| Skin rash | 8 | 6 |
| Light liver function impairment | 4 | 8 |
| Headache | 5 | 6 |
| Low blood pressure | 3 | 5 |
| Gastrointestinal bleeding | 4 | 4 |
| Palpitations | 3 | 4 |
| Gum bleeding | 3 | 2 |
