# Supplementary material for: The add-on effects of Danhong injection among patients with ischemic stroke receiving Western medicines: A systematic review and meta-analysis
Source: Front Pharmacol. 2022 Aug 23;13:937369. doi: 10.3389/fphar.2022.937369 (PMC9445550; doi:10.3389/fphar.2022.937369)
Supplement: Supplementary file 3 [file DataSheet2.DOCX]

**Appendix 2** Supplementary figures


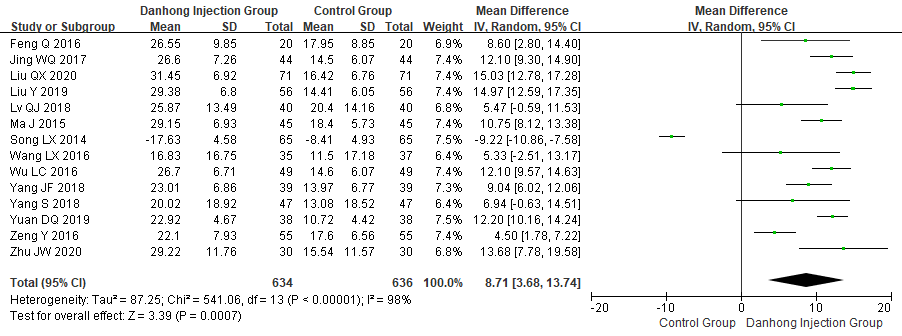


**Figure S1.** Meta-analysis of Barthel index between DHI+WM and WM in patients with Ischemic Stroke

**
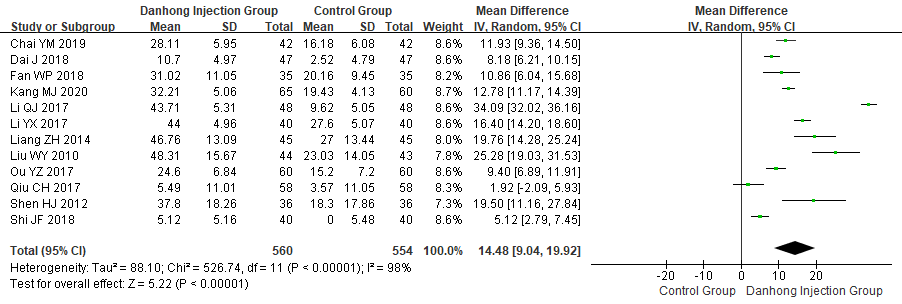
**

**Figure S2.** Meta-analysis of ADL between DHI+WM and WM in patients with Ischemic Stroke


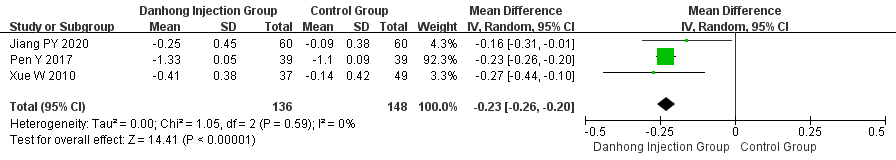


**Figure S3.** Meta-analysis of IMT between DHI+WM and WM in patients with Ischemic Stroke


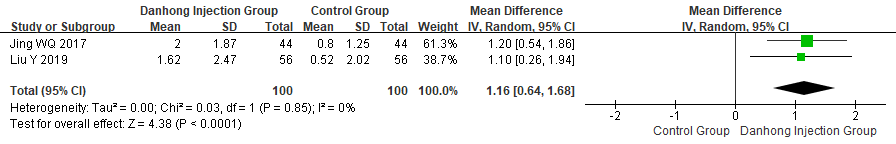


**Figure S4.** Meta-analysis of Cerebral Blood Flow between DHI+WM and WM in patients with Ischemic Stroke


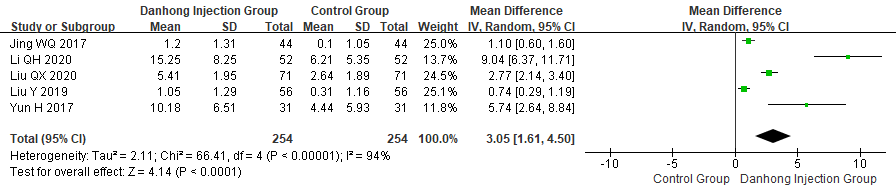


**Figure S5.** Meta-analysis of Average Cerebral Blood Flow Rate between DHI+WM and WM in patients with Ischemic Stroke


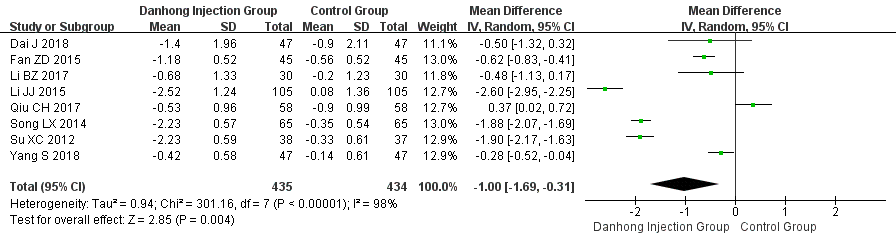


**Figure S6.** Meta-analysis of triglycerides between DHI+WM and WM in patients with Ischemic Stroke


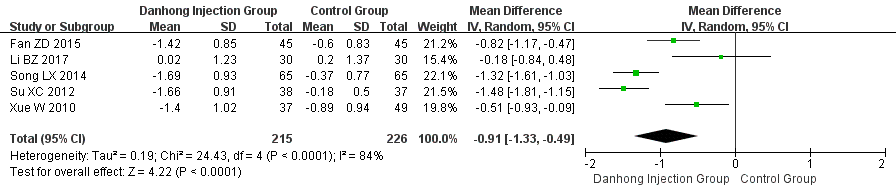


**Figure S7.** Meta-analysis of LDL between DHI+WM and WM in patients with Ischemic Stroke


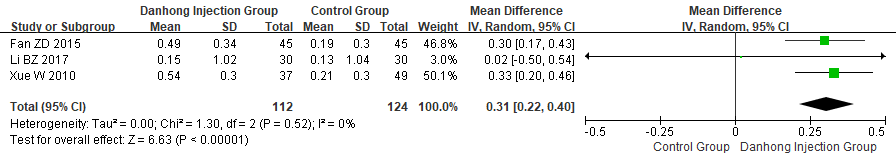


**Figure S8.** Meta-analysis of HDL between DHI+WM and WM in patients with Ischemic Stroke


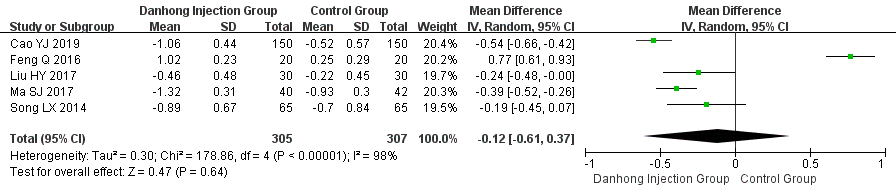


**Figure S9.** Meta-analysis of D-dimer between DHI+WM and WM in patients with Ischemic Stroke


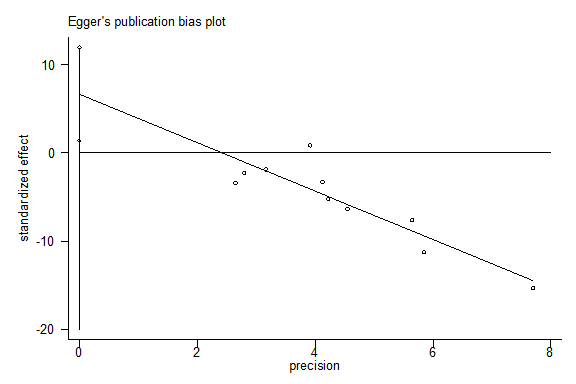


**Figure S10.** Egger’s publication bias plot of total cholesterol


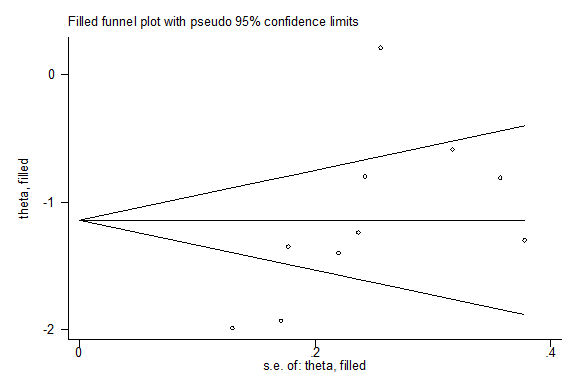


**Figure S11.** Filled funnel plot of total cholesterol from studies that investigated the association between the efficacy of DHI and WM. The circles are real studies and the trim-and-fill analysis estimating there were no possible missing studies. The horizontal line represents the summary effect estimates, and the diagonal lines represent pseudo-95% CI limits
