# Supplementary material for: The add-on effects of Danhong injection among patients with ischemic stroke receiving Western medicines: A systematic review and meta-analysis
Source: Front Pharmacol. 2022 Aug 23;13:937369. doi: 10.3389/fphar.2022.937369 (PMC9445550; doi:10.3389/fphar.2022.937369)
Supplement: Supplementary file 4 [file DataSheet1.DOCX]

**Appendix 1** Search strategies

1. **PubMed** **(**Search date: July 20, 2020**)**

#1 "stroke"[MeSH] OR "stroke"[Title/Abstract]

#2 "[cerebral infarction](https://www.ncbi.nlm.nih.gov/mesh/68002544)"[MeSH] OR "[cerebral infarction](https://www.ncbi.nlm.nih.gov/mesh/68002544)"[Title/Abstract]

#3 " brain infarction "[MeSH] OR " brain infarction "[Title/Abstract]

#4 " brain ischemia "[MeSH] OR " brain ischemia "[Title/Abstract]

#5 " cerebral ischemia "[Title/Abstract]

#6 " intracranial arterial disease "[MeSH] OR " intracranial arterial disease "[Title/Abstract]

#7 "cerebrovascular disorder "[MeSH] OR " cerebrovascular disorder "[Title/Abstract]

#8 " carotid artery disease "[MeSH] OR " carotid artery disease "[Title/Abstract]

#9 " intracranial embolism "[MeSH] OR " intracranial embolism "[Title/Abstract]

#10 #1 or #2 or #3 or #4 or #5 or #6 or #7 or #8 or #9

#11 danhong injection [Title/Abstract]

#12 #10 and #11

1. **Embase (Ovid) (**Search date: July 20, 2020**)**

1 stroke.mp. or stroke/

2 cerebral infarction.mp. or cerebral infarction/

3 brain infarction.mp. or brain infarction/

4 brain ischemia.mp. or brain ischemia/

5 cerebral ischemia.mp. or cerebral ischemia/

6 intracranial arterial disease.mp. or intracranial arterial disease/

7 cerebrovascular disorder.mp. or cerebrovascular disorder/

8 carotid artery disease.mp. or carotid artery disease/

9 intracranial embolism.mp. or intracranial embolism/

10 1 or 2 or 3 or 4 or 5 or 6 or 7 or 8 or 9

11 danhong injection.mp.

12 10 and 11

13 clinical trial.mp. or clinical trial/

14 random*.mp. or random*/

15 randomized controlled trial.mp. or randomized controlled trial/

16 13 or 14 or 15

17 12 and 16

1. **Cochrane Central Register of Controlled Trials** **(Ovid)** (Search date: July 20, 2020)

1 stroke.mp. or stroke/

2 cerebral infarction.mp. or cerebral infarction/

3 brain infarction.mp. or brain infarction/

4 brain ischemia.mp. or brain ischemia/

5 cerebral ischemia.mp. or cerebral ischemia/

6 intracranial arterial disease.mp. or intracranial arterial disease/

7 cerebrovascular disorder.mp. or cerebrovascular disorder/

8 carotid artery disease.mp. or carotid artery disease/

9 intracranial embolism.mp. or intracranial embolism/

10 1 or 2 or 3 or 4 or 5 or 6 or 7 or 8 or 9

11 danhong injection.mp.

12 10 and 11

1. **China National Knowledge Infrastructure Database (CNKI)** (Search date: July 20, 2020)

SU=('卒中'+'脑梗死'+'脑梗塞'+'脑缺血'+'脑血栓'+'脑栓塞'+'脑血管事件'+'中风'+'脑血管意外' ) AND SU='丹红注射液' AND 全文='随机'

1. **VIP Database** (Search date: July 20, 2020)

U=(卒中 OR 脑梗死 OR 脑梗塞OR 脑缺血 OR 脑血栓 OR 脑栓塞 OR脑血管事件 OR 中风 OR 脑血管意外) AND U=丹红注射液

1. **Wanfang database** (Search date: July 20, 2020)

主题:( "卒中" or "脑梗死" or "脑梗塞" or "脑缺血" or "脑血栓" or "脑栓塞" or "血管事件" or "中风" or "脑血管意外") and 主题:( "丹红注射液")and 主题:( "随机")
